# Maggot-associated *Ignatzschineria larvae* bacteremia: a case report

**DOI:** 10.1128/asmcr.00113-24

**Published:** 2025-03-25

**Authors:** Paolo Gigante, Gabriele Arcari, Donatella Ossola, Benedetta Pennella, Luigina Guasti, Federica Novazzi, Mattia Carbotti, Gianluca Cassani, Domenico Caleca, Riccardo Capuano, Renée Pasciuta, Nicasio Mancini

**Affiliations:** 1 Laboratory of Medical Microbiology and Virology, ASST Sette Laghi, University Hospital of Varese, Varese, Italy; 2 Department of Medicine and Technological Innovation, University of Insubria 19045 , Varese, Italy; 3 Department of Geriatrics, ASST Sette Laghi, Angera Hospital 472719 , Varese, Italy; 4 Department of Medicine, ASST Sette Laghi, University Hospital of Varese 472719 , Varese, Italy; 5 Department of Medicine and Surgery, University of Insubria 19045 , Varese, Italy; Rush University Medical Center, Chicago, Illinois, USA

**Keywords:** myiases, 16S rRNA

## Abstract

**Background:**

Myiases are fly larvae parasitic infections involving host tissues. In humans, myiases generally occur in people with reduced self-care capacity or living in poor hygienic conditions, with wounds serving as an entry point for flies. In addition to direct larvae damage, myiases constitute a risk factor for secondary bacterial infections.

**Case Summary:**

An 81-year-old patient with multiple comorbidities and living in poor hygienic conditions accessed the emergency room of a secondary care hospital in the Varese area following deterioration in his clinical condition and presence of maggots on his right leg. Blood cultures grew gram-negative bacilli, which could not be identified using standard procedures: biochemical tests yielded *Pasteurella canis* as a result with 95% probability, whereas matrix-assisted laser desorption ionization time-of-flight mass spectrometry could not find the spectrum in its database. The species *Ignatzschineria larvae* was determined by 16S rRNA sequencing, and its significance was interpreted upon consultation between microbiologists and clinicians.

**Conclusion:**

This case underscores the role of collaboration between clinical microbiology laboratories and clinicians in diagnosing and managing uncommon infections. The identification of *I. larvae* was obtained through 16S rRNA sequencing, highlighting how sequencing-based approaches are becoming increasingly implemented as diagnostic tools when traditional methods fail in identifying rare microorganisms.

## INTRODUCTION

Infestation of live vertebrates with Diptera larvae (maggots) is defined as myiases. According to the relationship between the parasite and the host, myiases can be defined as facultative, obligatory, or accidental (1). Facultative myiases are parasitic afflictions of humans or other animals caused by fly larvae colonizing necrotic or, less frequently, viable tissues (1). These infestations generally occur in individuals with poor hygiene or reduced self-care capacity, where open wounds serve as entry points for flies to deposit their larvae and can be classified as cutaneous, sanguinivorous, wound, and cavitary (1). Myiases have traditionally been associated with tropical and subtropical climates, but reports of these infestations in temperate regions have increased in recent years, especially in people living near livestock, which leads to increased exposure to parasitic flies (1).

In addition to the direct tissue damage caused by larvae, myiases pose a risk of secondary bacterial infections. Some fly species (and their larvae) can act as vectors for bacteria, which can be the cause of infection, notwithstanding their relatively low pathogenic potential. For this reason, the presence of myiases should alert healthcare providers to the possibility of concurrent bacterial infections that may extend beyond the infected tissue and involve systemic involvement.

This case report presents an 81-year-old man with a chronic, larvae-infested wound, which developed a bloodstream infection caused by *Ignatzschineria larvae*.

The *Ignatzschineria* genus was first described in 2001 as part of the microbiome of a Sarcophagidae fly, *Wohlfahrtia magnifica* (2), and named after the entomologist who first described *W. magnifica* (Ignaz Rudolph Schiner) (3, 4). As of September 2024, the genus *Ignatzschineria* includes a total of six species, five validly published and approved (*I. larvae*, *I. indica*, *I. ureiclastica*, *I. cameli*, and *I. rhizosphaerae*) and the Candidatus *Ignatzschineria merdigallinarum*. While the first isolation of all these species occurred from insects of the order Diptera, *Ignatzschineria* spp. isolates were also sampled from other specimens (e.g., livestock or fermented foods) (2).

Specifically, *I. larvae* is an aerobic, non-motile, non-spore-forming gram-negative bacillus (5). After overnight incubation on blood agar plates, *I. larvae* form small, convex, non-pigmented, and translucent colonies, while growth on MacConkey agar plates may be observable after 48–72 h of incubation. Since both biochemical- and mass spectrometry-based identification approaches may provide incorrect or no identification, nucleic acid sequencing is the only method allowing a reliable identification. Albeit having an environmental origin, *Ignatzschineria* isolates have been identified as incidental pathogens following maggot-related infections in both animals and humans (6–11).

Here, we describe a case of bacteremia caused by *I. larvae* in a patient with a chronic wound colonized by larvae.

## CASE PRESENTATION

An 81-year-old man living in poor hygienic conditions with a remote medical history characterized by obesity, dyslipidemia, asthma-related emphysematous chronic obstructive pulmonary disease, and chronic arterial hypertension accessed the emergency room of a secondary care hospital in the Varese area, Northwest Italy, close to the Swiss border because of a deterioration in his clinical conditions (day 0).

**Fig 1:**
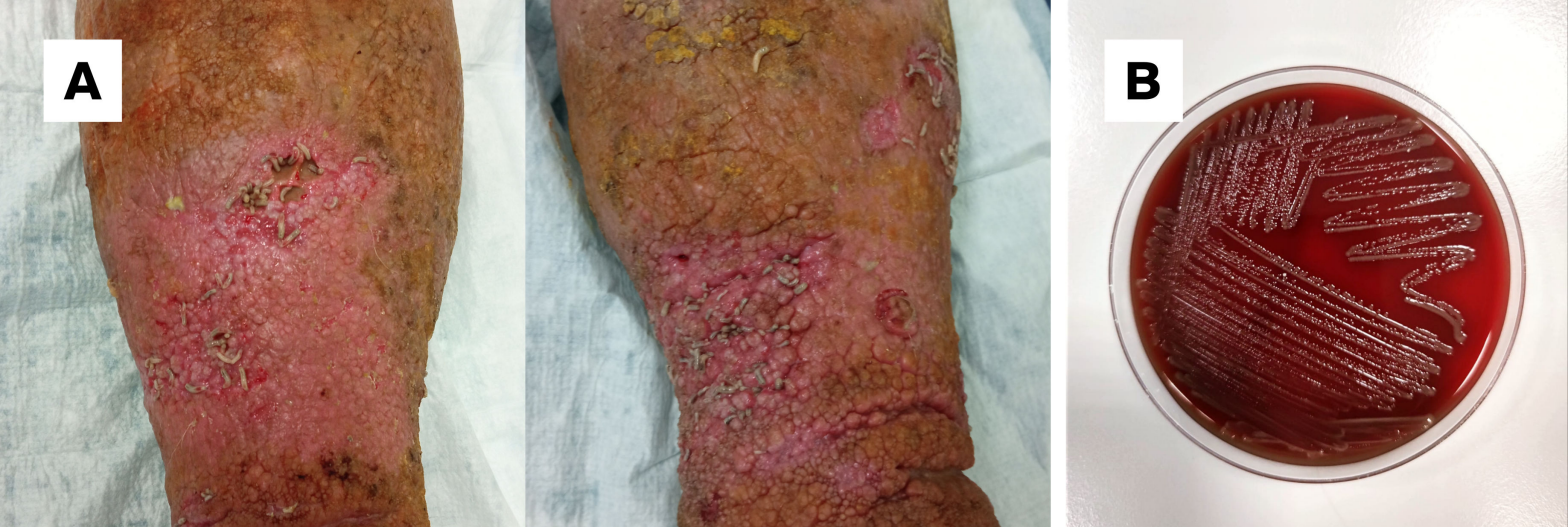
Maggot-infested wound before mechanical removal of the larvae. (B) Growth of *Ignatzschineria larvae* colonies on 5% sheep blood-enriched Columbia agar after 36 h of incubation at 37°C under aerobic conditions

Upon clinical observation, the patient was febrile (37.8°C) and showed an organized hematoma of the right elbow, lymphadenopathy of the lower limbs with infected ulcers on the right leg in the anterior tibial region associated with chronic lymphedema, positive Stemmer’s sign, hyperkeratosis, and lymphatic papillomatosis. Notably, the patient also presented with a chronic wound on the distal third of the right leg, showing extensive necrosis and the presence of moving larvae (Fig. 1A). At the same time, surgical debridement of the wound and mechanical removal of larvae were performed. Unfortunately, the larvae were not conserved, making species identification impossible.

**Fig 2:**
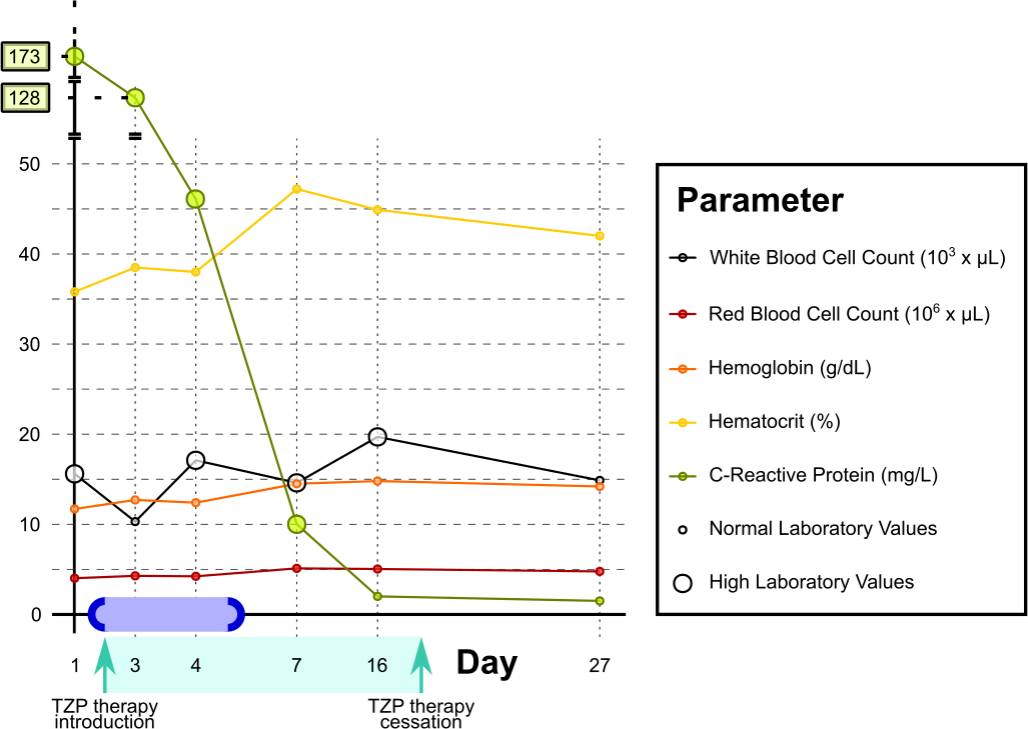
Timeline of the case study. Laboratory testing results represented sequentially according to patient timeline. Blood culture timing is illustrated as a light blue rectangle; the dark blue semicircle on the left of the rectangle designates the sampling date, while the dark blue semicircle on the right of the rectangle designates the positivity date. Some blood laboratory values not presenting at any point any difference from reference values (i.e., platelets, creatinine, natremia, and kalemia) were not reported for clarity’s sake.

Admission laboratory tests revealed an elevated white blood cell count (15,580/mm³) and C-reactive protein (173 mg/L). On day 2, blood cultures were collected, and antibiotic therapy with piperacillin–tazobactam (TZP) was initiated (Fig. 2; Table 1).

**TABLE 1:** Antimicrobial resistance profile of the *Ignatzschineria larvae* isolate

Class	Antimicrobial agent	MIC (µg/mL)
**Beta-lactams**	Aztreonam	≤1
	Ceftolozane/tazobactam	≤0.25
	Piperacillin/tazobactam	≤4
	Cefepime	≤1
	Ceftazidime/avibactam	≤0.25
	Meropenem	≤0.12
	Imipenem	≤1
	Meropenem/vaborbactam	≤0.06
	Imipenem/relebactam	0.5
Tetracyclines	Tigecycline	≤0.5
	Eravacycline	>0.5
Aminoglycosides	Amikacin	≤2
	Tobramycin	≤0.5
Polymixins	Colistin	≤0.5

On day 5, a vascular surgeon evaluated the patient’s clinical picture and recommended continuing with wound dressings. A duplex ultrasound of the lower limbs excluded deep and superficial vein thrombosis but revealed bilateral saphenofemoral junction and left saphenopopliteal junction incompetence. Concurrently, out of the two blood culture sets collected at admission, one aerobic bottle turned positive after approximately 51 h. Preliminary Gram staining revealed gram-negative rods. After subculture and 36 h of incubation, abundant growth of small, translucent colonies was observed on Columbia agar with 5% sheep blood (Fig. 1B) and chocolate agar. Gram staining from colonies revealed gram-negative rods.

Matrix-assisted laser desorption ionization time-of-flight mass spectrometry (MALDI-TOF MS) (VITEK MS, bioMérieux, Marcy-l'Étoile, France, CE-IVD database for bacteria, version 3.3.1.1) could not identify the microorganism. Hence, after nucleic acid extraction, a V3–V4 16S rRNA variable region sequencing approach (Applied Biosystems 3500, Thermo Fisher Scientific, Waltham, MA) was adopted.

Nucleotide BLAST of the sequence in the rRNA/ITS database led to the identification of *I. larvae* (99.5% identity and one gap with GenBank accession number AJ252143).

Upon consultation between the clinical microbiology laboratory and the clinicians taking care of the patient, the presence of this microorganism was considered relevant since it perfectly matched the case presentation.

Antimicrobial susceptibility testing was performed using broth microdilution (Sensititre Gram-Negative MIC Plate, Thermo Fisher Scientific). European Committee on Antimicrobial Susceptibility Testing breakpoint tables for interpretation of MICs lack specific criteria for *I. larvae*. However, the broth dilution MIC for the TZP combination was ≤4 µg/mL; hence, according to the “When there are no breakpoints in breakpoint tables?” guidance document (12), therapy with TZP could have been considered and was, therefore, carried over.

Biochemical identification of the isolate yielded *Pasteurella canis* with 95% probability (VITEK2 GN identification card, bioMérieux) (Table 2). On blood agar plates, however, the isolate grew, forming convex, smooth, and opaque colonies (Fig. 1B), clearly differing from what is typically observed with *P. canis*. Hence, because the patient’s clinical conditions improved under TZP treatment, this preliminary identification was not shared with clinicians, and 16S sequencing was performed.

**TABLE 2:** Biochemical characteristics of the *Ignatzschineria larvae^a^*

Biochemical reaction	Result^*b*^	Biochemical reaction	Result^*b*^
APPA	-	lMLTa	-
H_2_S	-	lARL	-
BGLU	-	dGLU	-
ProA	-	dMNE	-
SAC	-	TyrA	-
lLATk	-	CIT	-
GlyA	-	NAGA	-
O129R	-	lHISa	-
ADO	-	ELLM	+
BNAG	-	dCEL	-
dMAL	-	GGT	-
LIP	-	BXYL	-
dTAG	-	URE	+
AGLU	-	MNT	-
ODC	-	AGAL	-
GGAA	-	CMT	-
PyrA	-	lLATa	-
AGLTp	-	BGAL	-
dMAN	-	OFF	-
PLE	-	BAlap	-
dTRE	-	dSOR	-
SUCT	-	5KG	-
LDC	-	PHOS	+
BGUR	-	Predicted species: *Pasteurella canis* (95%)	

^*a*^ APPA, alkaline phosphatase activity; H₂S, hydrogen sulfide production; BGLU, β-glucosidase activity; ProA, proline aminopeptidase activity; SAC, saccharose (sucrose) fermentation; lLATk, leucine arylamidase (leucine aminopeptidase); GlyA, glycine aminopeptidase; O129R, resistance to O/129 (Vibrio static agent); ADO, adonitol (ribitol) fermentation; BNAG, β-N-acetylglucosaminidase activity; dMAL, D-maltose fermentation; LIP, lipase activity; dTAG, D-tagatose fermentation; AGLU, α-glucosidase activity; ODC, ornithine decarboxylase activity; GGAA, glycylglycine aminopeptidase activity; PyrA, pyroglutamic acid aminopeptidase activity; AGLTp, agmatine utilization (agmatine deiminase pathway); dMAN, D-mannose fermentation; PLE, phospholipase activity; dTRE, D-trehalose fermentation; SUCT, succinic acid utilization; LDC, lysine decarboxylase activity; lMLTa, L-malate utilization; lARL, L-arabitol fermentation; dGLU, D-glucose fermentation; dMNE, D-mannitol fermentation; TyrA, tyrosine aminopeptidase activity; CIT, citrate utilization; NAGA, N-acetyl-D-glucosamine fermentation; lHISa, L-histidine assimilation; ELLM, Ellman’s reaction (thiol production); dCEL, D-cellobiose fermentation; GGT, gamma-glutamyltransferase activity; BXYL, β-xylosidase activity; URE, urease activity; MNT, mannitol fermentation; AGAL, α-galactosidase activity; CMT, α-C-methyl-D-glucoside fermentation; lLATa, L-leucine aminopeptidase activity; BGAL, β-galactosidase activity; OFF, oxidation/fermentation of carbohydrates; BAlap, beta-alanine aminopeptidase activity; dSOR, D-sorbitol fermentation; 5KG, 5-Keto-D-gluconate utilization; PHOS, phosphatase activity; BGUR, β-glucuronidase activity

^*b*^ -, negative; +, positive.

During the 4 weeks of hospitalization, the patient underwent significant variations in his therapy compared with his previous home care (i.e., diuretic, antihypertensive, corticosteroid, and insulin replacement therapy, as well as a cholesterol-lowering treatment) and went through a physical rehabilitation course. Moreover, owing to the patient’s social vulnerability, the case was referred to social workers, and the patient was transferred to a subacute care unit to maintain glycemic control in recently diagnosed diabetes, optimize circulatory compensation, and continue wound care for the lower limbs.

## DISCUSSION

Our case report of a documented instance of *I. larvae* bacteremia in Italy underscores the diagnostic and clinical challenges associated with uncommon bacterial infections if not properly correlated with the clinical picture. The patient presented with chronic larva-infested wounds and posed a microbiological conundrum when conventional diagnostic methods yielded inconclusive or misleading results. In contrast to published reports (13), *I. larvae* was not identified by MALDI-TOF MS analysis. The biochemical identification testing predicted the isolate as *P. canis* with a high probability, a misclassification that could have led to inappropriate treatment and misinterpretation of the clinical scenario. Definitive identification was achieved only through 16S rRNA gene sequencing, emphasizing the need to implement this approach into clinical microbiology laboratories to address the limitations of traditional identification methods for rare or unconventional pathogens.

Additionally, this case draws attention to bacterial infections associated with facultative myiases, underlying the need for greater knowledge of the range of bacteria involved in facultative and obligatory myiases and their clinical implications. Colonized wounds create an environment conducive to secondary infections by various microorganisms, including rare pathogens (14, 15).

Furthermore, the case highlights the critical importance of close clinical laboratory collaboration. In the absence of clinical information, it would not have been feasible to link the isolated microbe to the patient’s condition, allowing for a precise diagnosis and successful treatment. Together, these elements were pivotal in achieving a favorable clinical outcome in a socially vulnerable patient with complex health needs.

## Data Availability

Assembled 16S rRNA bacterial gene amplicons based on the V3–V4 region sequencing are available in NCBI under accession number PQ892126.1.
